# Unveiling the Identity of *Viburnum laterale* (Viburnaceae), a Long‐Lost Species Rediscovered After Nearly a Century, Based on Morphological and Molecular Evidence

**DOI:** 10.1002/ece3.73517

**Published:** 2026-05-12

**Authors:** Liao‐Cheng Zhao, Wen‐jun Lyu, Hong‐tao Liu, Ming Tang

**Affiliations:** ^1^ College of Forestry Jiangxi Agricultural University Nanchang Jiangxi China; ^2^ College of Ecology and Nature Conservation Beijing Forestry University Beijing China; ^3^ National Germplasm Repository of Viburnum, Wuhan Botanical Garden, Chinese Academy of Sciences Wuhan Hubei China; ^4^ CAS Key Laboratory of Aquatic Botany and Watershed Ecology, Wuhan Botanical Garden Chinese Academy of Sciences Wuhan Hubei China; ^5^ Hubei Key Laboratory of Wetland Evolution and Ecological Restoration, Wuhan Botanical Garden Chinese Academy of Sciences Wuhan Hubei China; ^6^ University of Chinese Academy of Sciences Beijing China; ^7^ Jiangxi Provincial Key Laboratory of Conservation Biology (2023SSY02081) Jiangxi Agricultural University Nanchang Jiangxi China; ^8^ Lushan National Observation and Research Station of Chinese Forest Ecosystem Jiujiang Jiangxi China

**Keywords:** China, taxonomy, *Viburnaceae*, *Viburnum*

## Abstract

*Viburnum laterale* Rehder is a poorly known shrub endemic to southeastern China that has remained taxonomically unresolved since its publication more than a century ago. The species was known only from a few historical collections that lacked critical characters, and no living populations had been documented for nearly a century. Here, we report the rediscovery of this taxon and clarify its taxonomic identity using an integrative approach combining morphological and molecular evidence. Observations of living plants reveal several previously undocumented reproductive traits, including inflorescences with conspicuous white sterile marginal flowers, as well as fruit and seed morphology. Comparative analyses of living material, type specimens, and protologues indicate that *V. laterale* is most similar to *V. hanceanum* Maximowicz, with *V. laterale* differing mainly in its nearly absent indumentum, longer peduncles, and more coarsely serrate leaf margins. Phylogenetic analyses based on chloroplast coding sequences, together with an expanded dataset comprising nuclear ribosomal internal transcribed spacer (nrITS) and three plastid markers (*mat*K, *ndh*F, and *rbc*L), consistently place *V. laterale* within sect. *Tomentosa* (Maximowicz) Nakai, where it forms a strongly supported clade with *V*. *hanceanum*. Based on morphological and molecular evidence, *V. laterale* is transferred to sect. *Tomentosa* and treated as a variety of *V*. *hanceanum*, namely *V. hanceanum* var. *depilatum* M. Tang & L.C. Zhao. Our study resolves a long‐standing taxonomic problem and highlights the importance of rediscovering historically ambiguous taxa for accurate species delimitation in *Viburnum*.

## Graphical abstract

Viburnum laterale, a poorly known species endemic to southeastern China, is rediscovered after nearly a century and reassessed using integrated morphological observations and molecular phylogenetic analyses. Newly documented reproductive traits and multilocus phylogenies place this taxon within Viburnum sect. Tomentosa, where it forms a strongly supported clade with *V. hanceanum*. Based on combined evidence, *V. laterale* is reduced to varietal rank as *V. hanceanum* var. *depilatum*, resolving a long‐standing taxonomic uncertainty.

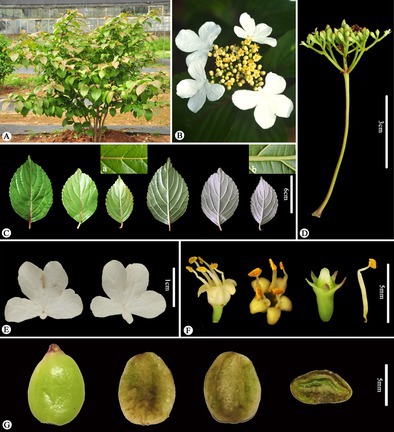

## 1 Introduction

*Viburnum* L. (Linnaeus 1753) comprises approximately 200 species of shrubs and small trees, widely distributed from subtropical regions of the Northern Hemisphere to the Andes and tropical Asia. China represents a major center of diversity for this genus, harboring 73 species, of which 45 are endemics (Yang and Malécot 2011). Originally classified within Caprifoliaceae (Cronquist 1968; Hsu 1988), subsequent phylogenetic studies based on morphological traits and molecular data have demonstrated that *Viburnum* is more closely related to genera currently assigned to Adoxaceae (Backlund and Bremer 1997). This treatment has since been widely accepted in recent classification systems (APG III 2009; Yang and Malécot 2011; APG IV 2016; Zhao et al. 2025). Although *Viburnum* has often been treated within Adoxaceae in recent classifications, Viburnaceae is adopted here following the nomenclatural treatment of Reveal (2008) and the International Code of Nomenclature for algae, fungi, and plants (Turland et al. 2018). Despite extensive taxonomic studies on *Viburnum* (Donoghue et al. 2004; Clement and Donoghue 2011, 2012; Clement et al. 2014; Landis et al. 2021), species delimitation within the genus remains challenging in China, largely due to incomplete morphological data and limited phylogenetic sampling (Zhao et al. 2025).

**FIGURE 1:**
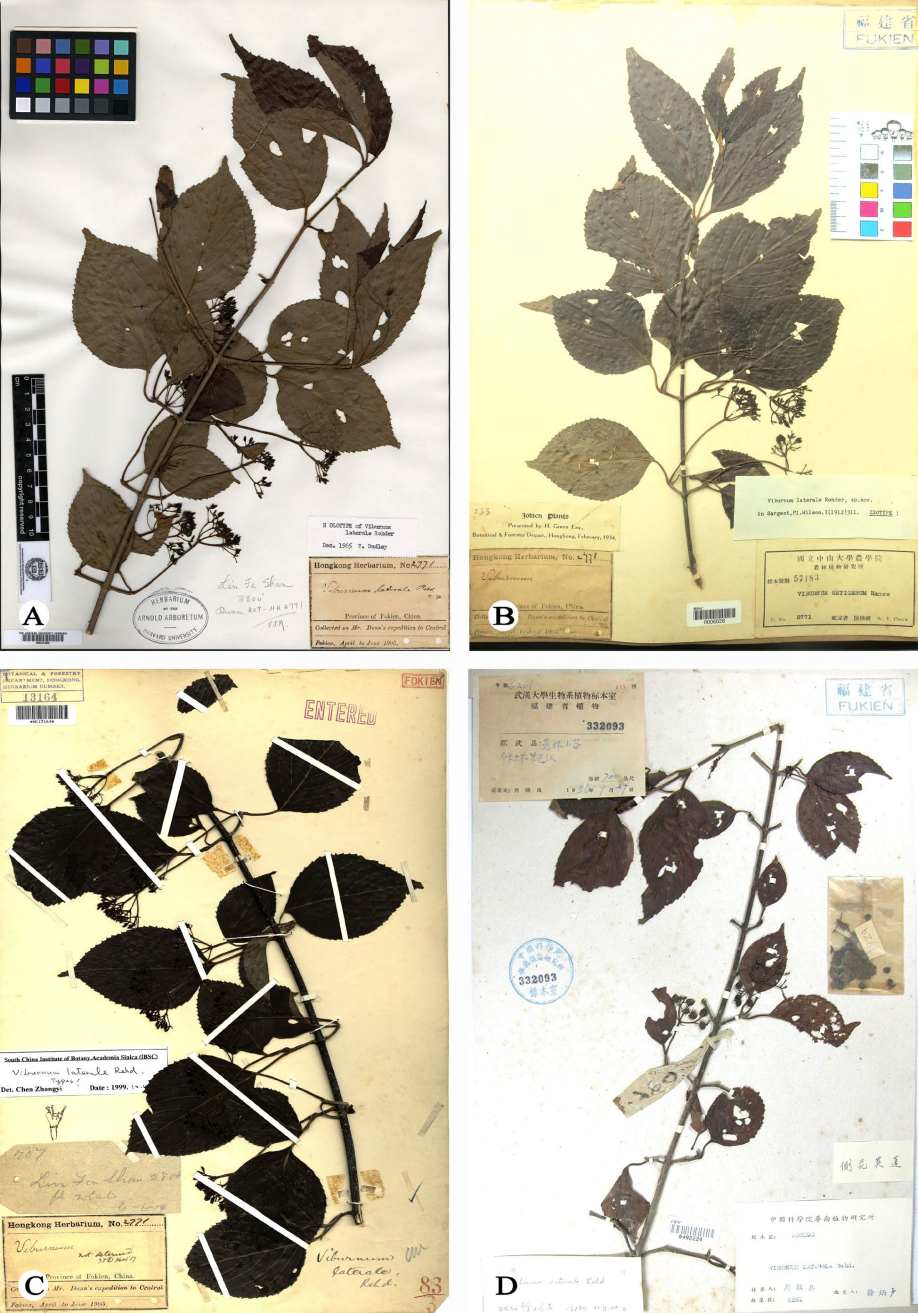
*Viburnum laterale*. (A) Fujian, China, *S.T. Dunn s.n*. (A00031569, holotype of *V*. *laterale*) (B) Fujian, China, *S.T. Dunn s.n*. (IBSC0006026, isotype of *V*. *laterale*) (C) Fujian, China, *S.T. Dunn s.n*. (HK0013164, isotype of *V*. *laterale*) (D) Shaowu city, Fujian, China, *H.C. Chou 6261* (IBSC0492224, *V*. *laterale*).

**FIGURE 2:**
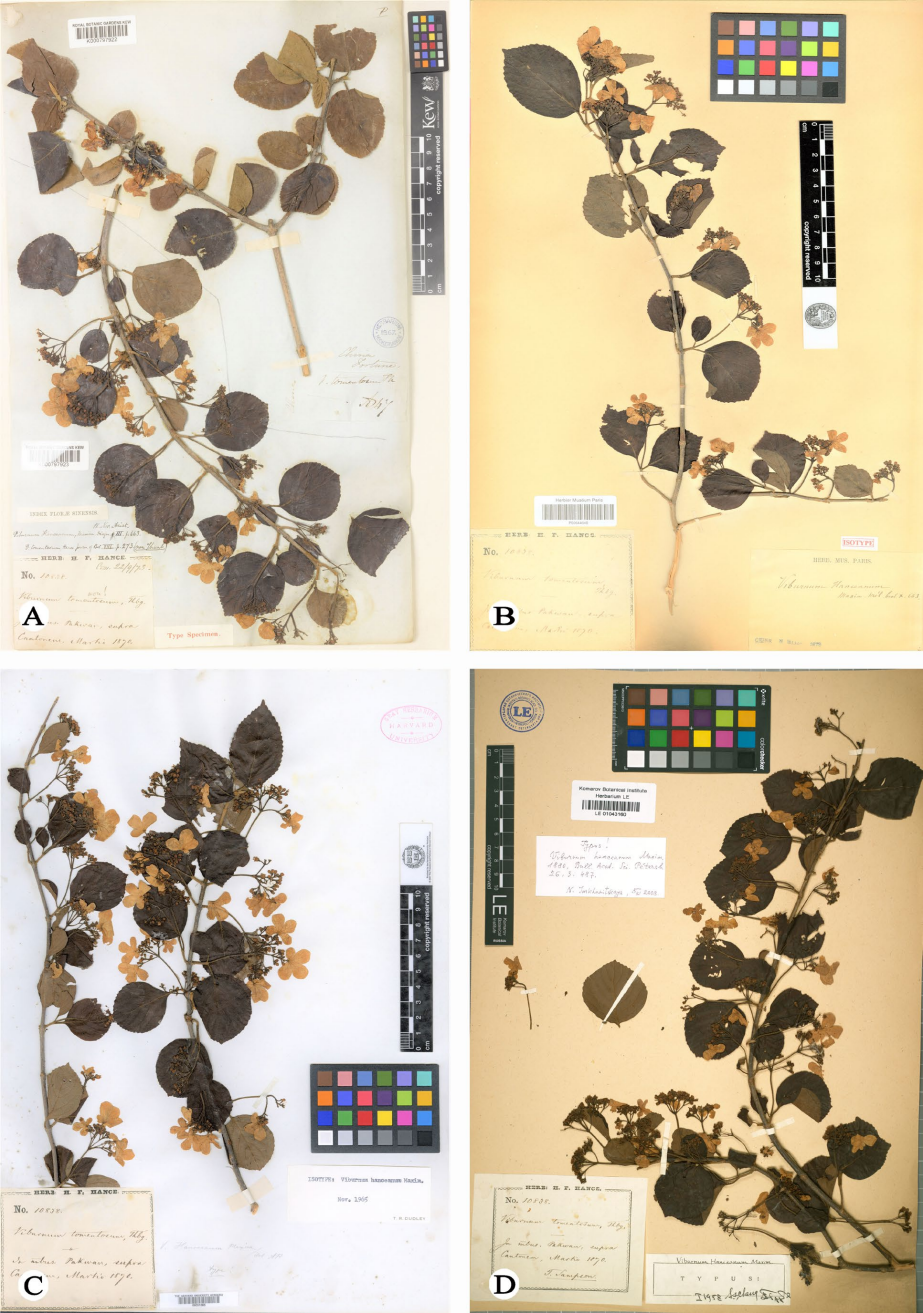
*Viburnum hanceanum*. (A) Baiyun Mountain, Guangzhou City, Guangdong, China, *G.T. Sampson s.n*. (K000797923, holotype of *V. hanceanum*) (B) Baiyun Mountain, Guangzhou City, Guangdong, China, *G.T. Sampson s.n*. (P00644640, isotype of *V. hanceanum*) (C) Baiyun Mountain, Guangzhou City, Guangdong, China, *G.T. Sampson s.n*. (GH00031565, isotype of *V. hanceanum*) (D) Baiyun Mountain, Guangzhou City, Guangdong, China, *G.T. Sampson s.n*. (LE01043160, isotype of *V. hanceanum*).

*Viburnum laterale* Rehder was first collected by S.T. Dunn during an expedition to central Fujian Province, China, in 1905 (Dunn 1908), and was formally described by Alfred Rehder based on the specimen *S.T. Dunn s.n*. (A) (Rehder 1911; Figure 1A). Since its original description, *V. laterale* has been rarely collected and remains poorly understood. The type material consists solely of immature fruiting specimens, lacking floral characters. As a result, its taxonomic status has been unstable, and the species has been variously assigned to sect. *Platyphylla* (Hsu) and sect. *Megalotinus* (Maximowicz) Rehder in subsequent treatments (Hsu 1988; Yang and Malécot 2011). Notably, a fruiting specimen collected from Shaowu, Fujian Province, in 1936 (*H.C. Chou 6261*; Figure 1D) showed striking morphological similarity to *V. hanceanum* (Maximowicz 1880; Figure 2A–D), a member of sect. *Tomentosa*, differing primarily in its nearly absent indumentum. However, the absence of flowering material and living populations has precluded a definitive assessment of their taxonomic relationship.

In 2021, during a botanical survey in Jiangshi Nature Reserve, Shaowu City, Fujian Province, China (27°04′44″N, 117°13′37″E), we discovered a living population of *Viburnum* matching the type locality and vegetative morphology of *V. laterale*. These shrubs are 1–2 m tall, absent indumentum, with inflorescences bearing a prominent outer ring of white sterile marginal flowers, the latter feature previously unrecorded for this species. We collected flowering individuals and introduced them into cultivation at the National Germplasm Repository of *Viburnum*, Wuhan Botanical Garden, Chinese Academy of Sciences, for further observations of fruit and seed morphology.

This rediscovery provides a rare opportunity to reassess the identity and taxonomic status of *V. laterale* using complete morphological information and molecular data. Specifically, our objectives were to (1) document previously unknown characters of *V. laterale*, (2) determine its phylogenetic position within *Viburnum*, and (3) reassess its taxonomic status.

## 2 Material and Methods

### 2.1 Morphological Observations

Living plants of *Viburnum laterale* were observed and photographed during the flowering in Jiangshi Nature Reserve, Fujian Province, China, and at the fruiting stage from individuals cultivated at the National Germplasm Repository of *Viburnum*, Wuhan Botanical Garden, Chinese Academy of Sciences. Detailed observations focused on inflorescence architecture, the presence and morphology of sterile marginal flowers, and fruit characters. All major morphological features of *V. laterale* and *V. hanceanum* were documented on living plants using a digital camera (Olympus TG‐6, Tokyo, Japan).

### 2.2 Taxon Sampling, DNA Extraction, and Data Collection

Taxon sampling followed previous phylogenetic studies of *Viburnum* (Donoghue et al. 2004; Clement and Donoghue 2011, 2012; Clement et al. 2014; Landis et al. 2021; Zhao et al. 2025) and the taxonomic framework proposed by Clement and Donoghue (2011). For analyses based on chloroplast coding sequences (CDS), 29 accessions representing 26 species and one variety from eight sections of *Viburnum* were sampled. To further ensure phylogenetic robustness, an expanded dataset was assembled using four commonly employed markers—three plastid markers (*mat*K, *ndh*F, *rbc*L) and the nrITS, including 62 accessions representing 57 species and three varieties across 10 sections within this genus. *Sambucus canadensis* L., *Adoxa moschatellina* L., *Tetradoxa omeiensis* (Hara) C.Y. Wu, and *Sinadoxa corydalifolia* C.Y. Wu, Z.L. Wu & R.F. Huang were selected as outgroups. Newly generated sequences were generated from three accessions of *V. laterale*, whereas all remaining sequences were downloaded from GenBank (https://www.ncbi.nlm.nih.gov/). Voucher information and GenBank accession numbers for all samples are provided in Tables S1 and S2.

Total genomic DNA was extracted from silica gel–dried leaf material of *V. laterale* using a modified CTAB protocol (Doyle and Doyle 1987). DNA integrity was assessed by 1% (w/v) agarose gel electrophoresis, and DNA quality and concentration were measured using a NanoDrop 2000 spectrophotometer (Thermo Scientific, Waltham, MA, USA). Library preparation and sequencing were performed by Novogene Co. Ltd. (Beijing, China). Paired‐end libraries (2 × 150 bp) were constructed following the Nova‐PE150 strategy, generating 2 Gb of raw sequencing data per sample.

Raw reads were filtered using Trimmomatic v.0.39 (Bolger et al. 2014) to remove adapter sequences, low‐quality bases and unpaired reads. Filtered reads were assembled with GetOrganelle v.1.7.7 (Jin et al. 2020), yielding complete chloroplast (cp) genomes and nuclear ribosomal DNA (nrDNA) sequences. Assembly graphs were visualized and manually inspected using Bandage v.0.8.1 (Wick et al. 2015). Chloroplast genome annotation was conducted in CPGAVAS2 (Shi et al. 2019) using *V. japonicum* (Thunberg) C.K. Sprengel (GenBank accession OP644292) as a reference. The sequences of plastid markers (*mat*K, *ndh*F, *rbc*L) were extracted from annotated cp genomes using Geneious Prime 2020 (Kearse et al. 2012), and nrITS sequences were extracted from nrDNA assemblies with ITSx (Bengtsson et al. 2013).

### 2.3 Phylogenetic Analysis

Sequence alignments were generated using MAFFT v.7.450 (Katoh and Standley 2013) with default parameters. Maximum likelihood (ML) analyses were conducted in IQ‐TREE (Nguyen et al. 2015), with nodal support assessed using 100,000 ultrafast bootstrap replicates. The best‐fit nucleotide substitution models were selected under the Bayesian Information Criterion (BIC) using ModelFinder (Kalyaanamoorthy et al. 2017). Bootstrap support values (MLBS) ≥ 70% were considered strongly supported (Huelsenbeck and Hillis 1993). Resulting phylogenetic trees were visualized and edited in FigTree v.1.4.4 (http://tree.bio.ed.ac.uk/software/figtree/).

## 3 Results

### 3.1 Morphological Study

**FIGURE 3:**
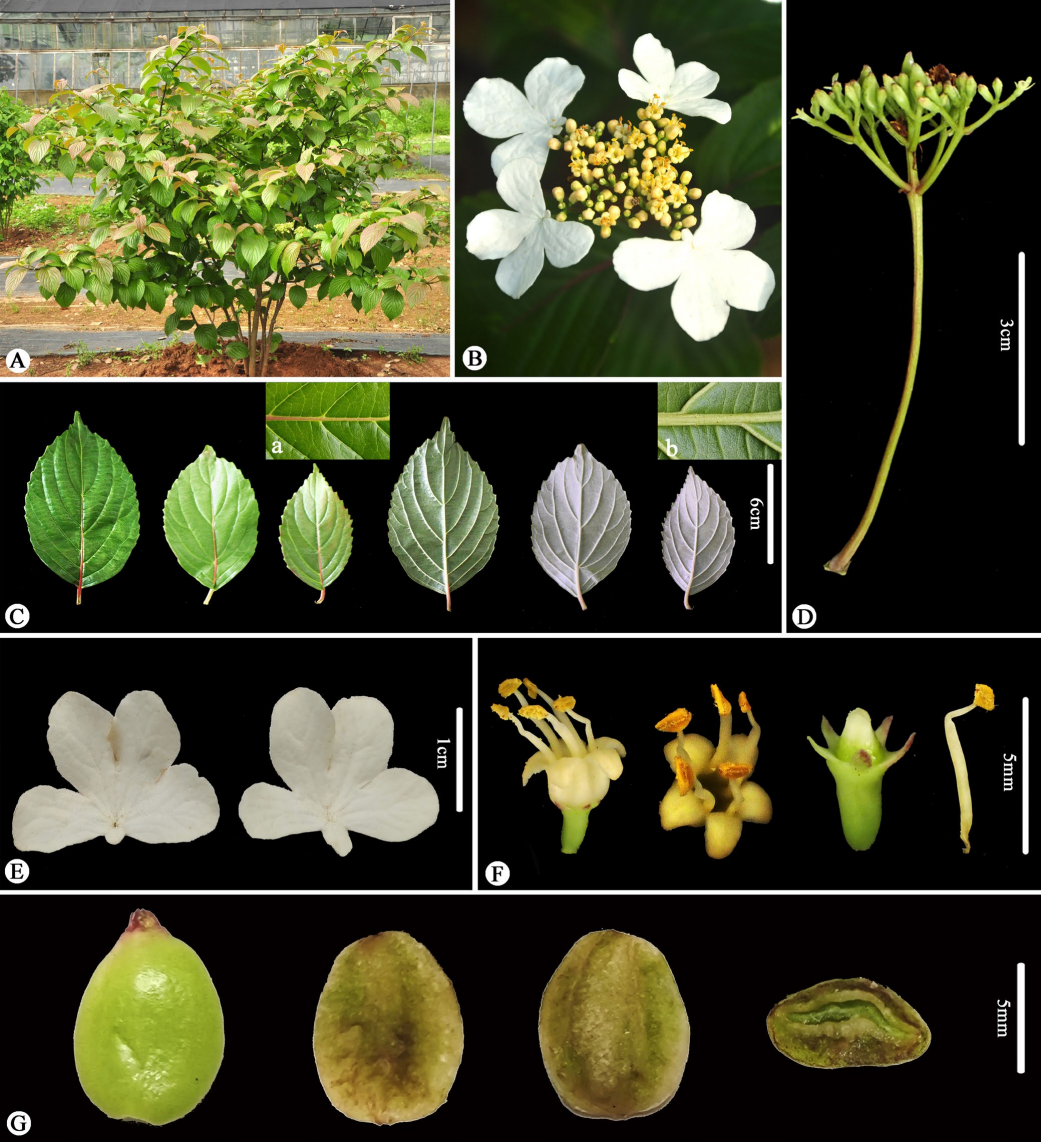
Morphology of *Viburnum laterale*. (A) habit (B) inflorescence (top view) (C) leaves (glabrous on both adaxial (a) and abaxial (b) sides) (D) inflorescence (side view) (E) white sterile flowers from the outer part of the inflorescence (F) from left to right: Side and top views of a fertile flower, stigma, and stamen, respectively (G) fruit (the left one) and seed (the right three).

Observations of living plants reveal several reproductive features of *Viburnum laterale* that were previously undocumented (Figure 3). The inflorescences are compound corymbs with a conspicuous outer ring of white sterile marginal flowers, surrounding numerous small yellow fertile flowers. Each fertile flower bears five stamens, a short style, and a capitate stigma. Fruits are ovoid drupes, turning from green to red and finally black at maturity, and seeds show two longitudinal grooves on the dorsal surface and a single groove on the ventral surface.

**FIGURE 4:**
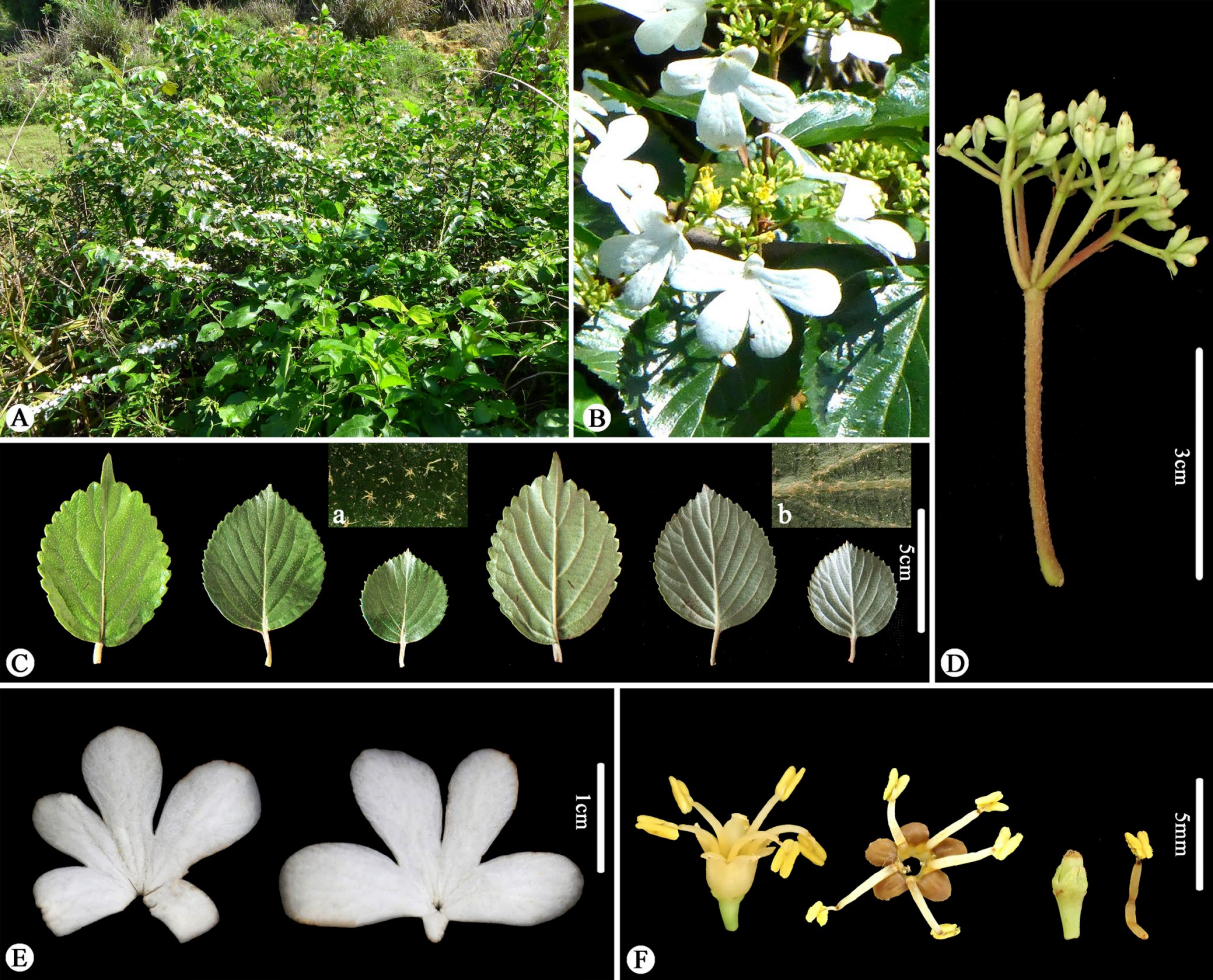
Morphology of *Viburnum hanceanum*. (A) habit (B) inflorescence (top view) (C) leaves (yellow‐brown fasciculate stellate‐pubescent on both adaxial (a) and abaxial (b) sides) (D) inflorescence (side view) (E) white sterile flowers from the outer part of the inflorescence (F) from left to right: Side and top views of a fertile flower, stigma, and stamen, respectively.

Comparisons based on specimens and living plants (Figures 1, 2, 3, 4) indicate that *V. laterale* and *V. hanceanum* share a highly similar inflorescence architecture, including the presence of white sterile marginal flowers, typically five primary rays, and fertile flowers borne on the third‐order branches. The two taxa differ mainly in vegetative characters. *V. laterale* is nearly glabrous throughout, with longer peduncles and more coarsely serrate leaf margins than those observed in *V. hanceanum* (Table 1).

**TABLE 1:** Morphological comparison of *Viburnum hanceanum* var. *depilatum* and *Viburnum hanceanum*.

Character	*Viburnum hanceanum* var. *depilatum*	*Viburnum hanceanum*
Branchlets	Current year branchlets brownish, glabrous; second year branchlets gray‐brownish, glabrous	Current year branchlets rusty stellate‐pubescent; second year branchlets purple‐brown, sparsely pubescent or subglabrous
Winter buds	Ovoid‐lanceolate, with 2 pairs of separate scales; scales glabrous	Lanceolate‐triangular, with 1 pair of adnate scales; scales yellowish brown stellate‐pubescent
Leaf	6–12 × 3–6 cm; glabrous; margin coarsely serrate	4–8 × 2.5–5.5 cm; yellow‐brown stellate‐pubescent; margin serrate
Peduncle	6–9 cm; glabrous	2–4 cm; yellow‐brown stellate‐pubescent
Fruit color	From green to red and turn black at maturity	From green to red at maturity
Seed dorsal grooves	2 dorsal grooves	1–3 dorsal grooves

### 3.2 Phylogenetic Study

**FIGURE 5:**
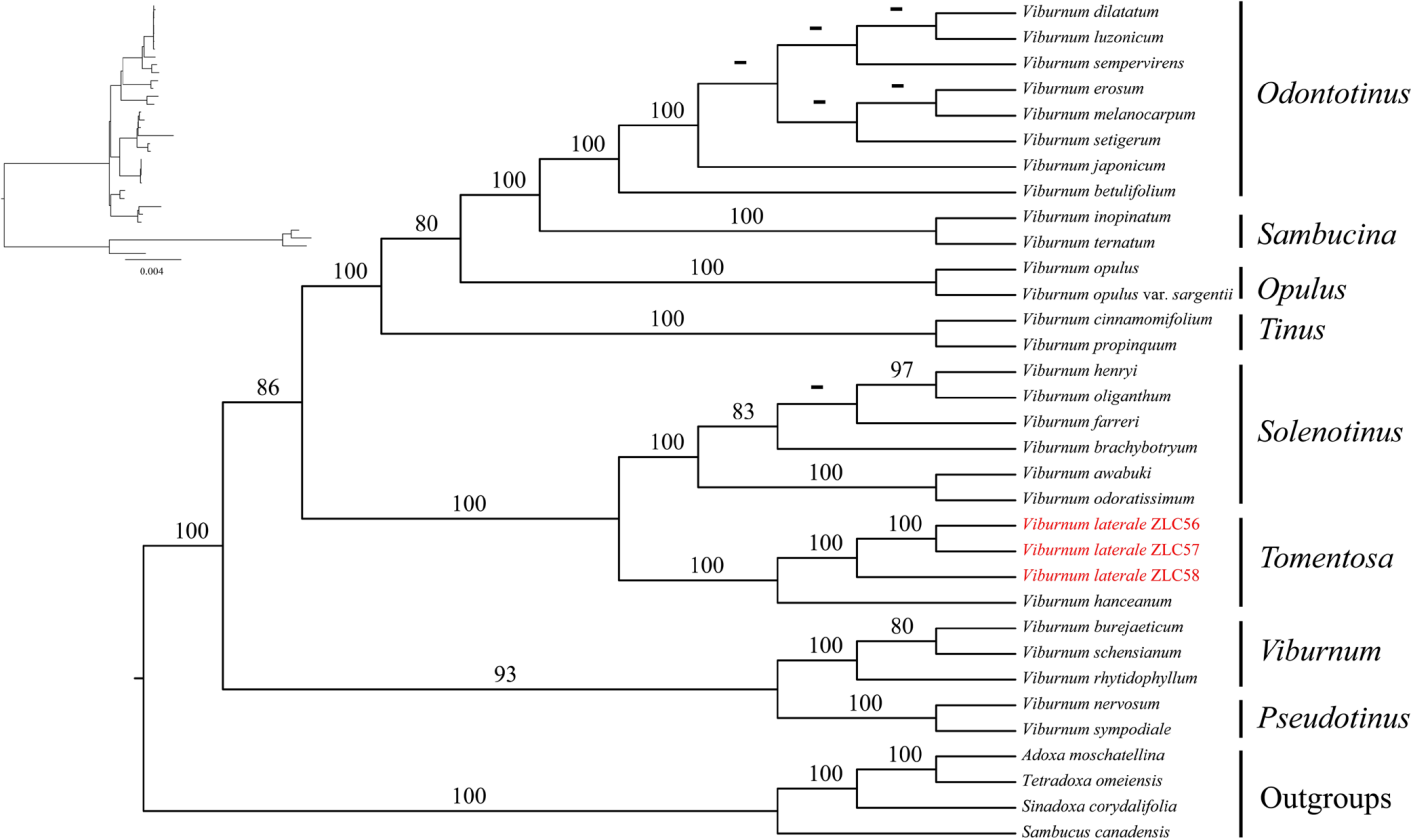
The maximum likelihood tree of *Viburnum* based on chloroplast coding sequence (CDS), with three *V. laterale* samples highlighted in red font. Bootstrap values (BS) are shown above the branches. Dashes (−) indicate MLBS < 70.

Maximum likelihood analysis based on chloroplast coding sequences (CDS) (best‐fit BIC model = TVM + F + I + R3) strongly supports the monophyly of *Viburnum* and its eight major clades (MLBS = 100; Figure 5). Within this framework, the three accessions of *V. laterale* form a well‐supported clade with *V. hanceanum* (MLBS = 100), together constituting sect. *Tomentosa*.

**FIGURE 6:**
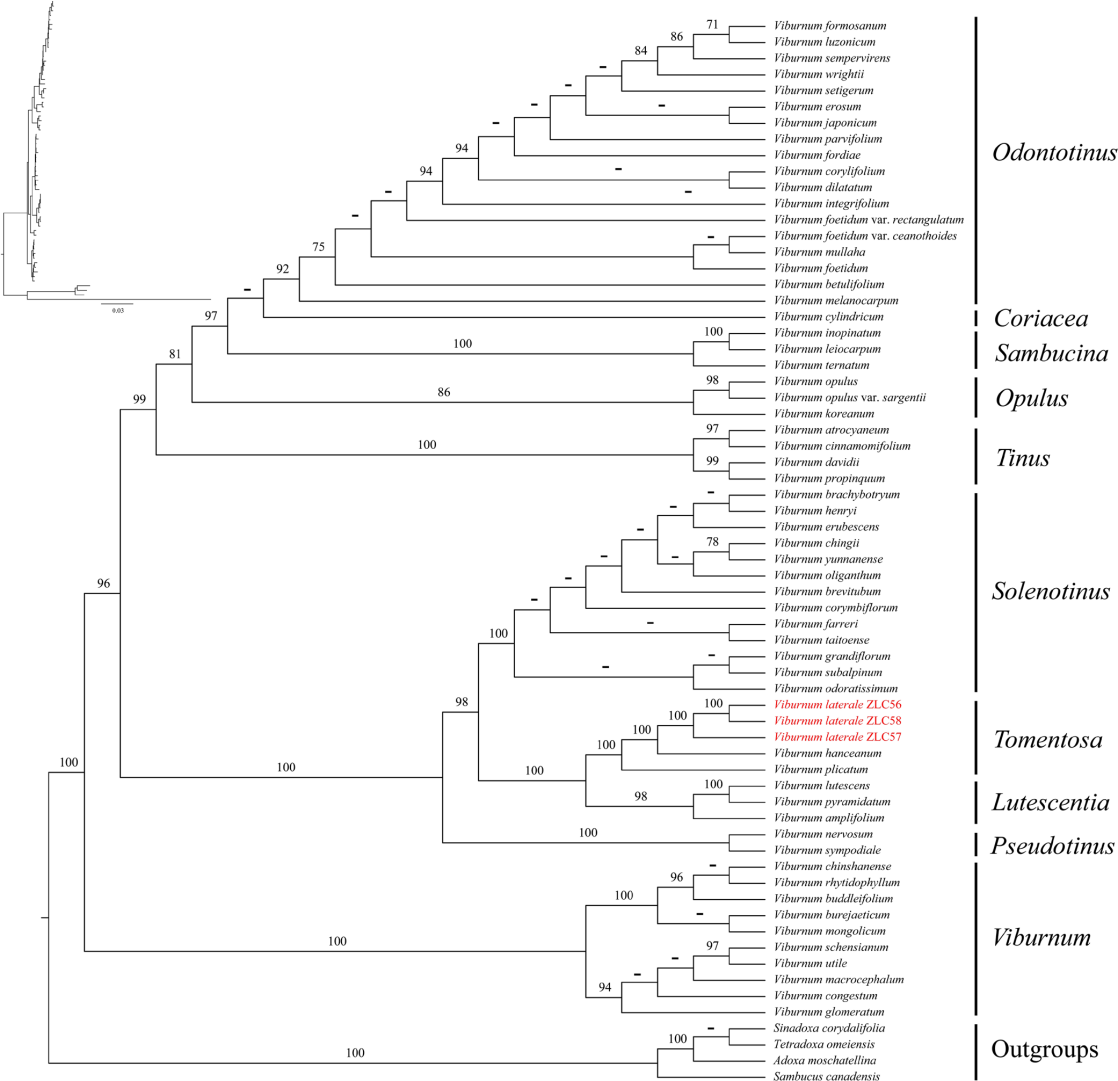
The maximum likelihood tree of *Viburnum* based on nrITS, *rbc*L, *mat*K and *ndh*F, with three *V. laterale* samples highlighted in red font. Bootstrap values (BS) are shown above the branches. Dashes (−) indicate MLBS < 70.

Analysis based on an expanded dataset combining *ndh*F, *ma*tK, *rbc*L, and nrITS (best‐fit BIC model = TPM3u + F + R2) yields a congruent topology (Figure 6). *Viburnum laterale* neither clusters with *V. amplifolium* Rehder, despite being placed in sect. *Platyphylla* by P.S. Hsu (1988), nor associates with the four sections segregated from sect. *Megalotinus* by Clement and Donoghue (2011): sect. *Coriacea* Kern, sect. *Sambucina* Kern, sect. *Lutescentia* Kern, and sect. *Punctata* Kern. Instead, *V. laterale* consistently forms a strongly supported clade with *V. hanceanum* (MLBS = 100; Figure 6).

### 3.3 Taxonomic Treatment

***Viburnum hanceanum* var. *depilatum* M. Tang & L.C. Zhao, var. nov**. (Figure 1A–C).

**Type:—**CHINA. Fujian province. Without precise locality, collection date unknown, *S.T. Dunn s.n*. (holotype: A00031569; isotype: IBSC0006026, HK0013164) (Figure 1A–C).

 = *Viburnum laterale* (1911: 311), **syn. nov**.

Type:**—**the type locality of *Viburnum laterale* is the same as above.

**Diagnosis**. *Viburnum hanceanum* var. *depilatum* is most closely allied to *V. hanceanum* in inflorescence architecture, particularly in having corymbose inflorescences with conspicuous white sterile marginal flowers, but differs from its nearly glabrous habit (vs. branchlets, petioles, peduncles, and leaves densely stellate‐pubescent), longer peduncles (6–9 cm vs. 2–5 cm), and more coarsely serrate leaf margins (Table 1 and Figures 1, 2, 3, 4).

**Description**. Shrubs, 1–2 m tall. Branchlets slender, terete, green to grayish brown, glabrous or very sparsely pubescent when young. Winter buds naked. Leaves opposite, petioles 1–3 cm long, glabrous; leaf blades ovate to elliptic, 6–12 cm long, 3–6 cm wide, papery to thinly coriaceous, both surfaces glabrous, lateral veins 6–8 pairs, impressed adaxially, prominent abaxially, base broadly cuneate to rounded, margin coarsely serrate, apex acuminate. Compound corymbs terminal, 5–9 cm in diameter, usually with five primary rays; peduncles slender, 6–9 cm long, glabrous. Marginal flowers sterile: calyx green, glabrous; corolla conspicuously enlarged, white, irregularly 4– or 5–lobed, lobes broadly ovate, corolla 2–3 cm in diam.; stamens and pistils absent or vestigial. Inner flowers fertile, bisexual; corolla white, rotate, ca. 3 mm in diam., glabrous. Stamens 5; style short; stigma capitate. Fruits drupaceous, ovoid, 6–8 mm long, ca. 4 mm wide, turning from green to red and finally black at maturity, pyrenes compressed. Seeds with two longitudinal grooves on the dorsal surface and one groove on the ventral surface.

**Phenology**. Flowering from April to May; fruiting from July to September.

**Distribution and habitat**. Endemic to southeastern China, currently known only from the type locality in Fujian Province. Grows in valleys, along streams, and in bushes at elevations of ca. 200–800 m a.s.l.

**Etymology**. The varietal epithet *depilatum* refers to the nearly absent indumentum of this taxon, in contrast to the densely pubescent typical variety of *Viburnum hanceanum*. We therefore propose the Chinese name ‘无毛蝶花荚蒾 (wú máo dié huā jiā mí)’.

**IUCN Red List Category**:—*Viburnum hanceanum* var. *depilatum* is currently known only from its type locality. Only one small population of this species, comprising fewer than 50 individuals, has been discovered there. The habitat of this taxon is now well preserved. Further populations are expected to be discovered as botanical exploration of northwestern Fujian province continues. According to the IUCN Red List Categories and Criteria (IUCN 2022), this taxon is best categorized as Data Deficient (DD).

**Additional specimens examined:** CHINA. Fujian Province, Shaowu City, Jiangshi Nature Reserve, 11 May 2024, *L. C. Zhao 56* (JXAU, paratype), *L. C. Zhao 57* (JXAU, paratype), *L. C. Zhao 58* (JXAU, paratype).

**Notes**. Morphological and molecular evidence supports the treatment of *Viburnum laterale* as an infraspecific taxon (variety) of *V. hanceanum*. The diagnostic characters distinguishing *V. hanceanum* var. *depilatum* and *V. hanceanum* (namely indumentum reduction, peduncle elongation, and leaf margin serration) are consistent across both wild and cultivated individuals in *V. hanceanum* var. *depilatum*, reflecting minor but stable vegetative differentiation below the species level.

## 4 Discussion

### 4.1 Resolving the Identity of a Long‐Overlooked Taxon

The rediscovery of *Viburnum laterale* after nearly a century has allowed a reassessment of a taxon whose identity had long been obscured by incomplete original material. The type specimen lacks flowers, which precluded the recognition of key reproductive traits and contributed to inconsistent sectional placements in earlier treatments (Hsu 1988; Yang and Malécot 2011). Observations of living plants reveal that *V. laterale* possesses inflorescences with conspicuous white sterile marginal flowers, as well as taxonomically informative fruit and seed features that were previously unavailable for evaluation.

Sterile marginal flowers are uncommon in Chinese *Viburnum* and are documented in only a limited number of taxa, spanning four section: sect. *Viburnum* (e.g., *V*. *keteleeri* Carrière, *V. macrocephalum* Fortune), sect. *Pseudotinus* C.B. Clarke (e.g., *V*. *sympodiale* Graebner), sect. *Opulus* (Miller) Candolle (e.g., *V. opulus* L, *V. opulus var. sargentii* Koehne), and sect. *Tomentosa* (e.g., *V. plicatum* var. *plicatum* Thunb., *V. hanceanum*, *V. hanceanum* var. *depilatum*, *V. plicatum* var. *formosanum* Y.C. Liu & C.H. Ou, and *V. plicatum* f. *tomentosum* (Miq.) Rehder). The scattered phylogenetic distribution of sterile marginal flowers suggests that this character has evolved independently multiple times within *Viburnum* (Park and Donoghue 2021). Although of limited diagnostic value at the sectional level, this character remains taxonomically informative at the species and infraspecific levels when considered in conjunction with vegetative morphology and molecular evidence.

### 4.2 Phylogenetic Position

Both morphological and molecular evidence places *V. laterale* within sect. *Tomentosa* (MLBS = 100; Figures 5 and 6), where it forms a strongly supported clade with *V. hanceanum* (MLBS = 100). This placement contrasts with earlier sectional assignments to sect. *Platyphylla* or sect. *Megalotinus* (Hsu 1988; Yang and Malécot 2011). Together, these results suggest that the misplacement of *V. laterale* in earlier treatments was likely a consequence of the absence of floral material in the type specimen (Figure 1A–C), rather than reflecting genuine phylogenetic affinity with those sections.

### 4.3 Taxonomic Status

Morphologically, *V. laterale* closely resembles *V. hanceanum*, particularly in inflorescence architecture and fertile flowers morphology. The two taxa differ primarily in vegetative traits, namely in indumentum, peduncle length, and the degree of leaf margin serration, all of which fall within the range of infraspecific variation commonly observed in *Viburnum*. Phylogenetic analyses consistently recover *V. laterale* as sister to *V. hanceanum* with strong support (MLBS = 100; Figures 5 and 6). Furthermore, based on currently available records, the known distribution of *V. laterale* falls entirely within the range of *V. hanceanum* (Hsu 1988; Yang and Malécot 2011). Taken together, this evidence suggests that the observed differences represent infraspecific variation rather than species‐level divergence. We therefore treat *V. laterale* as a variety of *V. hanceanum* and recognize it as *V. hanceanum* var. *depilatum* M. Tang & L.C. Zhao, var. nov.

## Author Contributions

**Liao‐Cheng Zhao:** conceptualization (supporting), formal analysis (lead), software (lead), visualization (lead), writing – original draft (lead), writing – review and editing (supporting). **Wen‐jun Lyu:** conceptualization (supporting), data curation (lead), investigation (lead), resources (supporting). **Hong‐tao Liu:** conceptualization (supporting), data curation (supporting), investigation (supporting), resources (supporting). **Ming Tang:** conceptualization (lead), formal analysis (supporting), funding acquisition (lead), methodology (supporting), project administration (lead), supervision (lead), writing – original draft (supporting), writing – review and editing (lead).

## Funding

This work was supported by the National Key R&D Program of China (2024YFF1307400) and the National Natural Science Foundation of China (grant No. 31960043).

## Ethics Statement

The authors have nothing to report.

## Conflicts of Interest

The authors declare no conflicts of interest.

## Supporting information


**Table S1:** Voucher information and GenBank accession numbers of complete chloroplast genome for all samples.

**Table S2:** Voucher information and GenBank accession numbers of ITS, matK, ndhF, rbcL for all samples.

## Data Availability

All newly generated DNA sequence data have been deposited in GenBank, and accession numbers are provided in Tables S1 and S2. Sequence alignments and phylogenetic trees supporting the conclusions of this study are available as Supporting Information—S1. All other data supporting the findings of this study are included within the article and its Supporting Information—S1.
